# Impulsive Action but Not Impulsive Choice Determines Problem Gambling Severity

**DOI:** 10.1371/journal.pone.0050647

**Published:** 2012-11-27

**Authors:** Damien Brevers, Axel Cleeremans, Frederick Verbruggen, Antoine Bechara, Charles Kornreich, Paul Verbanck, Xavier Noël

**Affiliations:** 1 Psychological Medicine Laboratory, CHU-Brugmann, Université Libre de Bruxelles, Bruxelles, Belgium; 2 Consciousness, Cognition and Computation (CO3), Université Libre de Bruxelles, Bruxelles, Belgium; 3 College of Life & Environmental Sciences, University of Exeter, Exeter, United Kingdom; 4 Department of Psychology, University of Southern California, Los Angeles, California, United States of America; 5 Clinical Research Division, Douglas Mental Health University Institute, Montreal, Canada; French National Centre for Scientific Research, France

## Abstract

**Background:**

Impulsivity is a hallmark of problem gambling. However, impulsivity is not a unitary construct and this study investigated the relationship between problem gambling severity and two facets of impulsivity: impulsive action (impaired ability to withhold a motor response) and impulsive choice (abnormal aversion for the delay of reward).

**Methods:**

The recruitment includes 65 problem gamblers and 35 normal control participants. On the basis of DSM-IV-TR criteria, two groups of gamblers were distinguished: problem gamblers (*n* = 38) and pathological gamblers (*n* = 27) with similar durations of gambling practice. Impulsive action was assessed using a response inhibition task (the stop-signal task). Impulsive choice was estimated with the delay-discounting task. Possible confounds (e.g., IQ, mood, ADHD symptoms) were recorded.

**Results:**

Both problem and pathological gamblers discounted reward at a higher rate than their controls, but only pathological gamblers showed abnormally low performance on the most demanding condition of the stop-signal task. None of the potential confounds covaried with these results.

**Conclusions:**

These results suggest that, whereas abnormal impulsive choice characterizes all problem gamblers, pathological gamblers' impairments in impulsive action may represent an important developmental pathway of pathological gambling.

## Introduction

For many people, gambling represents an occasional and recreational activity (e.g., usual lottery players). However, for some people it may become detrimental and evolve in an addiction that is a burden (e.g., work, family and financial problems). Once addicted, gamblers persist in playing for many “good” overt and covert reasons. However, like in other addictions, negative consequences directly associated with gambling seem to be weak regulators of these behaviors.

As one possible explanation, addiction to gambling may be due to poor control of impulses [1]. For instance, poor inhibition of prepotent responses in adult outpatient pathological gamblers may contribute to pathological gambler's weak capacity to remain abstinent 1 year after being enrolled in cognitive behavioral treatment for their pathological gambling problem [2]. Diminished self-regulation is reflected in the inability to inhibit the salient response associated with gambling, either cognitively (e.g., positive expectations to gamble), emotionally (e.g., sadness, excitement), or behaviorally (e.g., keep gambling). Highlighting the importance of poor self-regulatory processes in the maintenance of abnormal gambling behaviors is consistent with modern theoretical proposals of loss of willpower in addiction [3], [4]. According to these authors, a state of addiction reflects an imbalance between sensitized automatic cognitive processes (e.g., memory bias for automatic activation of gambling-related associations, attentional biases for gambling cues) [5], [6] and hampered capacities of self-control (e.g., suppressing the prepotent response, resisting interference) [7]. This imbalance between strong automatic processes making gambling very ‘wanted’ and weak cognitive processes unable regulating them, could reflect a state of impulsivity.

In this view, “impulsivity” is an umbrella concept encompassing a great number of automatic (e.g., reward processing) and intentional mechanisms (e.g., effortful inhibition) [8]. In the present study, we focused on the distinction between two behavioral expressions of impulsivity; *impulsive action* and *impulsive choice* [9]–[11]. *Impulsive action* refers to the inability to withhold a response and thereby reflecting poor response inhibition. Other behaviors do not result from response inhibition deficits but reflect *impulsive choice*, that is, an abnormal level of delay aversion as exemplified by increased preference for immediate reward over more beneficial but delayed reward. This distinction has been justified by behavioral and neurobiological evidence supporting distinct cortico-striatal substrates [11]. For instance, *impulsive action* reflects a disorder of dysregulation of action associated with diminished inhibitory control, whereas *impulsive choice* could be a motivational style (delay aversion) associated with fundamental alterations in reward mechanisms [12]. Moreover, neither in rats nor humans was impulsive action related to impulsive choice [13].

Interestingly, both dimensions of impulsivity are associated with pathological gambling but their respective influences on facets of pathological gambling is not clear yet [14]. Thus, pathological gamblers (1) exhibit impaired response inhibition performance (i.e., increased action impulsivity) [7], [15]–[18] and (2) show a preference for small, immediately available rewards over a variety of larger but delayed rewards, including long term financial health (i.e., increased choice impulsivity) [19].

Impulsive action could be indexed by stop-signal [20], [21] and go/no-go tasks [22], which require the subject to withhold simple motor responses when a stop-signal occurs (stop-signal task) or when a no-go stimulus is presented (go/no-go task). Impulsive choice could be assessed through the delay discounting task (DDT) [23]. In a typical delay discounting procedure, participants are asked to make a series of choices between large rewards (e.g. $1000) delayed by various delay intervals (e.g. 6 h to 25 years) and smaller immediate rewards (e.g. $1–$999). At each delay, the magnitude of the small immediate reward is adjusted until the small immediate and large delayed rewards are subjectively equivalent in value, referred to as the indifference point.

However, despite the accumulating evidence supporting the view that impulsivity is not a unitary construct, to the best of our knowledge there is no data available on within-subject comparisons of various aspects of impulsivity in problem or pathological gambling. This within-subject approach is particularly suited to examine whether the multidimensional nature of impulsivity may help to discriminate gamblers varying in their degree of problem gambling severity. For instance, in nicotine dependence, initial level of nicotine self-administration is primarily associated with impulsive action whereas impulsive choice seems to be related to the persistence of nicotine seeking during abstinence together with an enhanced sensitivity to nicotine-associated cues in both humans [24] and rats [25]. Hence, these later findings indicate that the involvement of impulsive action and impulsive choice may vary across different stages or clinical manifestations of nicotine dependence. Despite the advantages of within-subject comparisons, association between problem gambling severity and impulsivity has been conducted in separate groups each performing impulsivity paradigms targeting impulsive action or impulsive choice independently. These studies demonstrated that problem gambling is positively associated with impulsive action [18] and impulsive choice [26], [27], but see [28]. However, because of major differences in the sample characteristics of these studies (i.e., duration of gambling practice, evaluation of gambling dependence severity, age, gender, depression, anxiety, psychiatric comorbidities), direct comparisons should be taken with caution. Hence, the current literature does not answer the question of which type of impulsivity (i.e., of action, of choice) characterizes individuals with problematic or pathological gambling, which is the aim of the present study.

Therefore, the aim of this study was to investigate the relationship between the severity of gambling dependence and two facets of impulsive control (i.e., impulsive action and impulsive choice) in a same study design. Severe pathological gamblers, at-risk gamblers, and non-gamblers were compared according to their cognitive self-regulation efficiency. Our hypotheses were (1) that pathological gamblers (PG) show more pronounced impulsive action and impulsive choice than problem gamblers (PrG), who would be more impulsive than non-gamblers, (2) that higher impulsivity in gambler group remains after controlling mood effects and IQ.

## Methods

### Ethics Statement

The ethical review board of the Brugmann Hospital approved the study and written informed consent was obtained from all participants.

### Participants and recruitment

**Figure 1:**
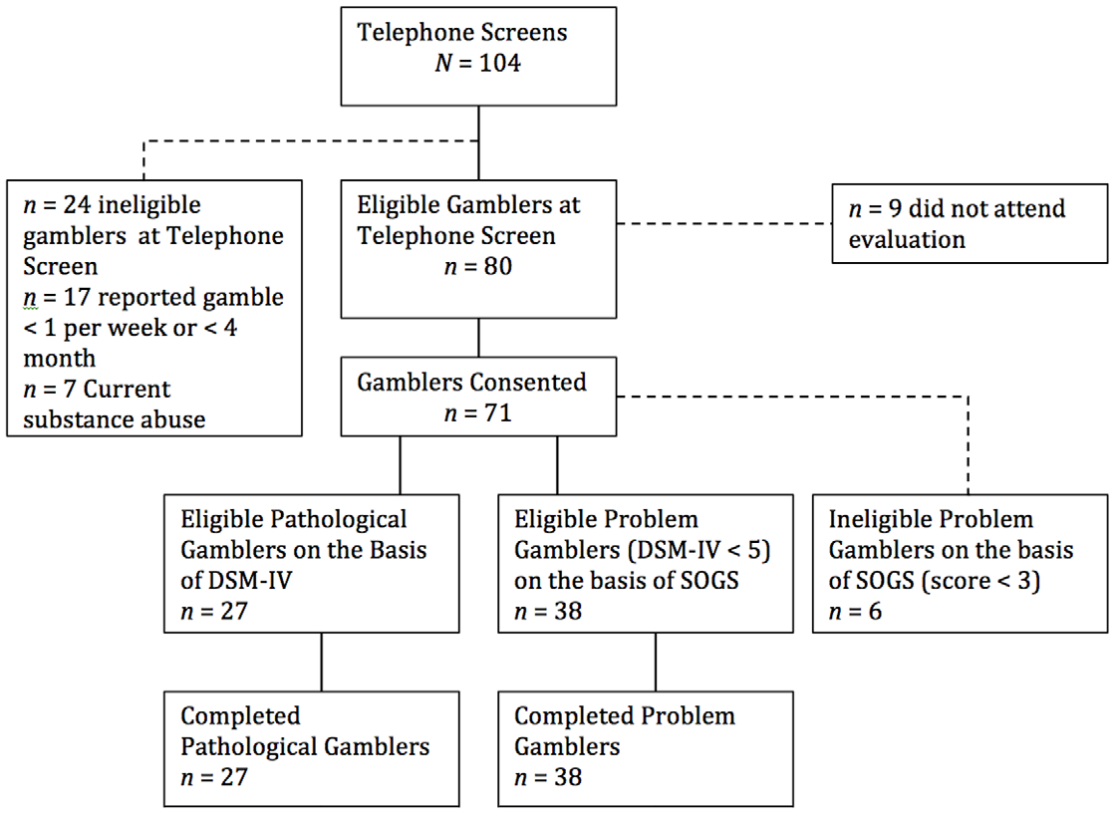
Flow of gamblers through the study.

Sixty-five gamblers and thirty-five non-gamblers participated in the study. Gamblers were recruited trough advertisement from the casino complex *VIAGE*, Brussels, Belgium. The ads asked for participants who “gambled frequently” to participate in a one-day study to explore factors associated with gambling. A telephone-screening interview was conducted by means of a locally developed screening tool, which included an examination of frequency of gambling behavior and comorbid psychiatric disorders. We excluded any subject who a) reported gambling in casino settings less than once a week or less than four times a month during the past 18 months, b) was older than 65 years (in order to avoid potential confounding from slow motor functioning due to aging), c) had experienced a substance use disorder during the year before enrollment into the study. In addition, participants were judged to be medically healthy on the basis of the results of their medical history. Substance use and medical history were examined on the basis of items taken from the Addiction Severity Index Short Form. The flow of gamblers through the study is presented in Figure 1.

Pathological gambling was assessed on the basis of the DSM-IV-TR. A total of 27 participating gamblers met the DSM-IV criteria for pathological gambling. Moreover, DSM-IV structured interview indicated that no gambler received therapeutic treatment at the time of the study. In addition, in order to distinguish frequent gamblers with a gambling problem from frequent gamblers without a gambling problem, gambling dependence severity was assessed using the South Oaks Gambling Screen (SOGS) [29]. Only 6 participants did not meet the criteria for low problem gambling (SOGS≥3). Due to statistical power issues, results of these 6 participants were not included in the present manuscript. All remaining gamblers (*N* = 65) met the criteria for low problem gambling, and 33 of those (51%) met the criteria for probable pathological gambling on the SOGS (SOGS≥5). We observed that none of the participants who scored 3 or 4 on the SOGS met the DSM-IV criteria for pathological gambling; 14 (70%) of the 20 respondents who scored 5, 6 or 7 on the SOGS met the DSM-IV criteria for pathological gambling; all of the 13 respondents who scored 8 or higher on the SOGS met the DSM-IV criteria for pathological gambling. Thus, a total of 27 pathological gamblers (PG) and 38 PrG participants were included in the study. Participants from the control group were recruited by word of mouth from the community (e.g., hospital employees). To avoid biases, resulting from inside knowledge of how these tasks operate, psychiatrists, psychologists and other personnel with psychological training were excluded from participation. The three groups were matched for age, gender, professional and educational level (see Table 1).

**Table 1:** Demographic and clinical information of participants.

	Normal Control	Problem Gamblers	Pathological Gamblers	Test Statistics	Bonferroni-corrected Pairwise Comparison
*n*	35	38	27		
Age (SD)	44.14(11.01)	38.46(15.42)	40.15(10.15)	*F*(2,97) = 1.88, *p* = .16	CONT = PG = PrG
Male/Female	28/7	28/10	21/6	X^2^(2,98) = 0.42, *p* = .81	CONT = PG = PrG
Employed full time % (*n*)	74.3(26)	71.05(27)	70.37(19)	X^2^(2,98) = 0.14, *p* = .93	CONT = PrG
Education% (*n*)					
<12th grade	34.3(12)	39.4(15)	40.7(11)	X^2^(2,98) = .33, *p* = .85	CONT = PrG
12th grade or higher	65.7(23)	60.6(23)	59.3(16)		
WAIS VOC	44.61(6.30)	45.60(6.15)	43.29(7.34)	*F*(2, 98) = 0.94, *p* = .39	CONT = PG = PrG
WAIS BLOC REP	15.51(1.89)	14.40(2.46)	14.18(2.88)	*F*(2, 98) = 1.71, *p* = .19	CONT = PG = PrG
WAIS BLOC TR	19.30(5.59)	21.24(6.92)	22.66(6.87)	*F*(2, 98) = 1.67, *p* = .27	CONT = PG = PrG
ADHD	7.61(3.31)	13.58(5.91)	12.52(3.79)	*F*(2, 97) = 15.23, *p*<.001	CONT<PG, PrG
BDI	2.29(2.47)	6.18(5.10)	10.44(6.02)	*F*(2, 98) = 31.63, *p*<.001	CONT<PrG<PG
STAI-S	30.29(9.96)	39.24(13.76)	43.78(13.72)	*F*(2, 98) = 8.23, *p*<.001	CONT<PrG, PG
STAI-T	36.64(7.31)	45.50(9.64)	49.73(9.53)	*F*(2, 98) = 17.76, *p*<.001	CONT<PrG, PG
SOGS	0.00(0.00)	4.69(2.88)	9.78(3.67)	*t*(65) = 6.39, *p*<.001	PrG<PG
Duration of gambling practice (in years)	**/**	17.53(12.87)	15.11(11.60)	*t*(65) = .65, *p* = .51	PrG = PG

Values shown are the mean and standard deviations on each measure. The South Oaks Gambling Screen was administered only in the PG and PrG groups. Degrees of freedom differ due to missing data. WAIS VOC = WAIS vocabulary, WAIS BLOC REP = WAIS block design correct responses, WAIS BLOC TR = WAIS bloc design reaction time, ADHD = attention deficit hyperactivity disorder, BDI = Beck Depression Inventory, STAI-S = State version of the State-Trait Anxiety Inventory, STAI-T = Trait version of the State-Trait Anxiety Inventory, SOGS = South Oaks Gambling Screen.

### Current clinical status

Current clinical status of depression and anxiety was rated with the Beck Depression Inventory [30] and the Spielberger State–Trait Anxiety Inventory (STAI) [31], respectively. Participants also completed the Adult ADHD Self-Report Scale (ASRS-v1.1) [32].

Intelligence was also estimated. We assessed intelligence with two subtests of the WAIS, namely the block design and vocabulary subtests [33]. This short form of the WAIS correlates with the full scale WAIS IQ in the 0.90 range [34].

### Impulsive choice measure: Delay Discounting Task (DDT)

The DDT [23] is a behavioral task that involves presenting participants a series of possible reward scenarios. The procedure used matched that of Madden and collaborators [23], who used the DDT with opiate addicts. Participants were repeatedly asked the question: “Would you rather have $X today, or $1000 in Y time period?” The task then systematically varied both the amount of money offered immediately and the length of time before receiving the delayed reward, permitting quantitative assessment of the function an individual uses to discount the reward based on its delay. Dollar (X) levels used were as follows: $1000, $990, $960, $940, $920, $850, $800, $750, $700, $650, $600, $550, $500, $450, $400, $350, $300, $250, $200, $150, $100, $80, $60, $40, $20, $10, $5, and $1. Time (Y) intervals used were 1 week, 2 weeks, 2 months, 6 months, 1 year, 5 years, and 25 years.

The DDT provides two reciprocal assessments. First, the individual was asked to choose between $1000 today or $1000 following the first time interval (one week). The immediate choice dollar value was then successively reduced until the participant switched their decision from selecting the discounted reward (e.g., $900) to the delayed full reward ($1000). Following the switch, the procedure continued for five additional monetary levels to ensure this was indeed the lowest point of choice modification. Seven trials of this procedure were employed, one for each of the time intervals. Second, following identification of the descending switch point for all seven-time intervals, the procedure began at the original time interval (one week) and was conducted in the opposite direction. That is, in the second part, the task began at the point five increments below the participant's switching point and successively increased the immediate reward until the individual stopped selecting the delayed reward ($1000) and returned to accepting the immediate reward. Similarly, the procedure continued for five additional reward levels beyond the switch to ensure this point was correctly identified. The dependent measure was constituted by the “point of indifference” which consisted of the average, for each time interval, of the descending and ascending switch points.

### Impulsive action measure: GoStop Impulsivity Paradigm (GoStop)

The GoStop was used to measure response inhibition [20]. In this task, participants were presented with a consecutive series of five-digit numbers on a computer screen. On each trial, novel stimulus (new, previously unseen set of five numbers) was presented for 500 ms, after which a target stimulus (a set of numbers always identical to the immediately preceding novel stimulus; in black font) appeared for 500 ms. Each trial was separated by a 1500 milliseconds interval. Participants were instructed to refrain from responding to the novel stimuli, and to click the mouse when presented with the black target (go) stimulus. However, on some trials, the black target (go) stimulus turned red (i.e., a ‘stop’ signal), which indicated that participants had to withhold their response to the target stimulus as well. Stop signals were presented 50 ms, 150 ms, 250 ms and 350 ms after the onset of presentation (stop-signal delays). The task was divided into two blocks of 80 trials. Participants were given a 30 seconds rest between the two blocks.

When the stop signal is presented shortly after the target stimulus, participants can easily withhold their response. As the delay between the target stimulus and the stop signal increases, probability of responding typically increases. To account for such observations, Logan and Cowan [35] proposed the *horse race model*, which assumes that two processes race against each other: a go process, triggered by the presentation of the target stimulus, and a stop process, triggered by the presentation of the stop signal. If the stop process finishes before the go process, participants inhibit their response; if the go process finishes before the stop process, response inhibition fails and subjects respond. In this study, we used percentage of inhibited responses (proportion of correctly inhibited responses to the number of stop signals presented) for each stop-signal delay (i.e., 50–350 ms) as a measure of response inhibition [36]. We expected that probability of responding would be higher in impulsive participants. Note that we did not have enough observations to reliably estimate the latency of the stop process [37].

### Procedure

This paper is part of a larger study into decision-making impairments in problem gambling. A paper regarding deficits of decision-making under uncertainty in problem gamblers was published elsewhere [38]. The current study focused on distinct facet of impulsivity in this same group of participants.

An intake interview was first undertaken, which included screening (in the gambler group: DSM-IV criteria for pathological gambling; in the control group: substance use and medical history of controls on the basis of items taken from the Addiction Severity Index Short Form) and self reports measures (SOGS score, current clinical status and demographics for all participants). Participants completed the computer task individually and in a quiet room, located at the Medical Psychology Laboratory, Brugmann Hospital. The order of test presentation was counterbalanced. No significant correlations between administration order and performance were present. After completion of the tasks, the research assistant answered any questions the participant had and provided payment. Participants received €15 for their participation.

### Data analysis

Initial data analysis involved assessing differences between groups on demographic variables (e.g., gender, age) and current clinical status (depression, anxiety, ADHD, IQ), using parametric or non-parametric statistics as appropriate.

Delay Discounting and GoStop tasks data were evaluated using repeated measures ANOVA. The nature of overall group effects and group by factor interactions was investigated using pairwise group comparisons investigating differences between pathological gamblers group (PG) and the control group, the PG group and the problem gambler (PrG) group, and the PrG group and the control group.

## Results

### Demographics and current clinical status

A description of demographic variables, scores on the South Oaks Gambling Screen (SOGS), estimated IQ, Adult ADHD Self-Report Scale (ASRS-v1.1), Beck's Depression Inventory (BDI) and the Trait and State version of the State-Trait Anxiety Inventory (STAI) is presented in Table 1. ANOVAs revealed that PG, PrG, controls were similar in terms of age, educational level, and estimated IQ (as measured by the Block Design and Vocabulary subtests of the Wechsler Adult Intelligence Test). Chi square analyses revealed no differences in the distribution of male and female participants. There was also no difference between PG and PrG on years of gambling use. There was a group difference on ADHD Rating Scale scores, *F*(2, 97) = 15.23, *p*<.001. Group contrasts revealed that PG and PrG groups scored higher than the control group (*p*<.001) on the ADHD Rating Scale. Depression was higher in PG than in PrG and in PrG compared to controls, *F*(2, 98) = 31.63, *p*<.001; contrasts: *p's*<.001. A group effect for State and Trait Anxiety was found, *F*(2, 98) = 8.23, *p*<.001; *F*(2, 98) = 17.76, *p*<.001, respectively. State and Trait anxiety were higher in the PG and in the PrG groups in comparison with the control group, *p's*<.001. No other group differences were present. We controlled for the potential covariate effect of ADHD, depression, and trait -state anxiety in subsequent analyses. Importantly, comparisons between PG, PrG and normal controls remained statistically significant when potentially confounding variables (ADHD, depression, trait and state anxiety) were individually entered as covariate into the statistical model.

### Impulsive choice: DDT

**Figure 2:**
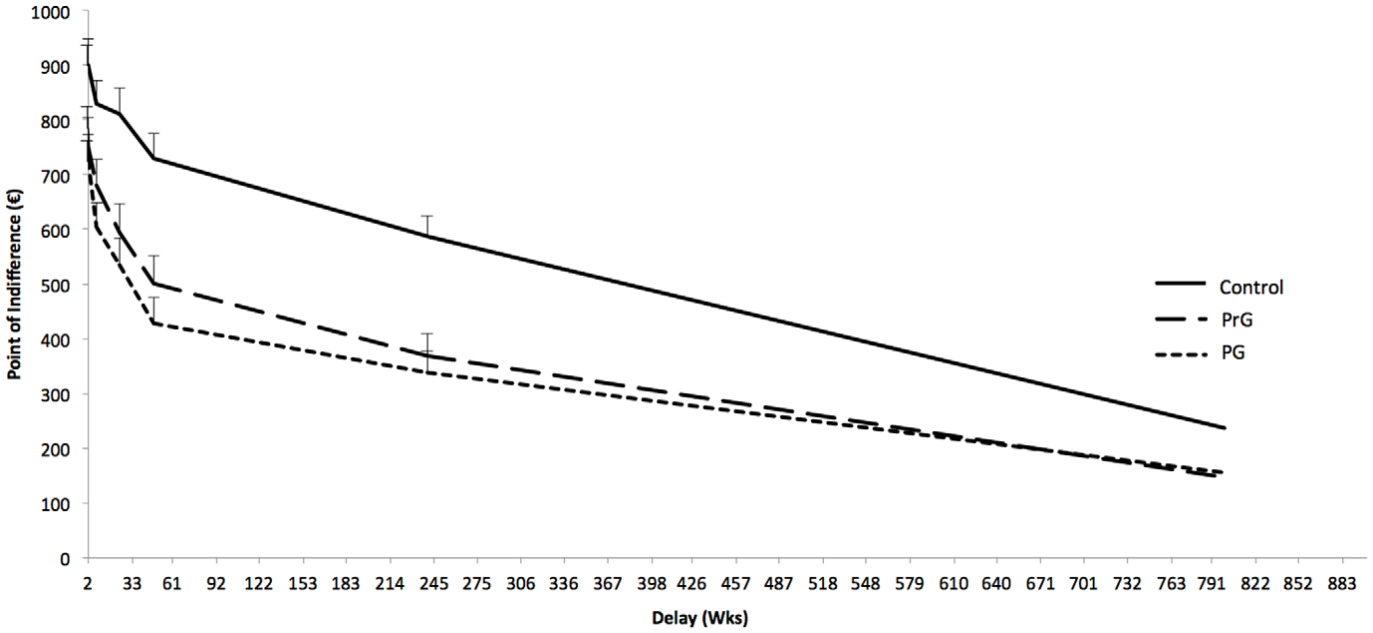
Delay discounting subjective euro amounts and delay periods. PrG = problem gamblers; PG = pathological gamblers.

Performance in the DDT shows that problem gamblers (PrG) and pathological gamblers (PG) discounted reward at a higher rate than control participants (see Figure 2). A repeated measures ANOVA was performed, with group as a between-subjects factor; time interval as a within subjects factor; and the point of indifference as the dependent measure. This analysis revealed an effect of time interval, *F*(2, 98) = 153.89, *p*<.001, η^2^ = .62, and a group effect, *F*(2, 98) = 9.95, *p*<.001, η^2^ = .18. Pairwise group comparisons revealed a significant difference between the PG and control group, *F*(1, 62) = 19.78, *p*<.001, η^2^ = .24, a significant difference between the PrG and control participants, *F*(1, 71) = 13.47, *p*<.001, η^2^ = .16, but no significant difference between the PG and PrG samples, *F*(1, 64) = 0.62, ns.

### Impulsive action: GoStop

**Figure 3:**
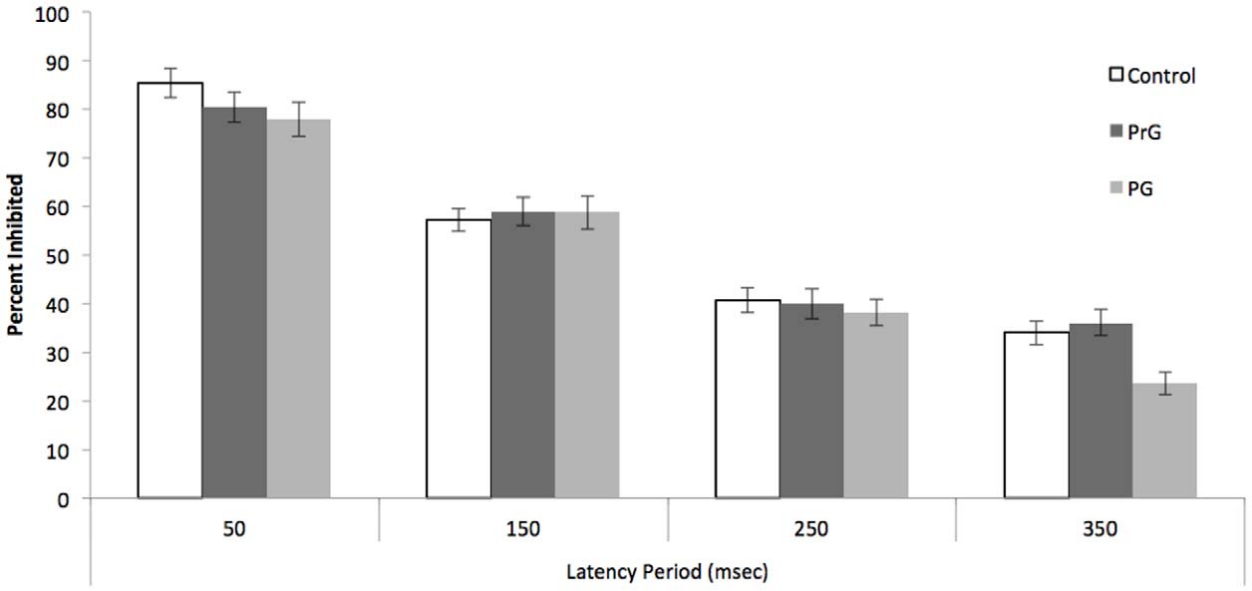
Mean of percent response inhibition at four stop-signal delays. PrG = problem gamblers; PG = pathological gamblers.

**Figure 4:**
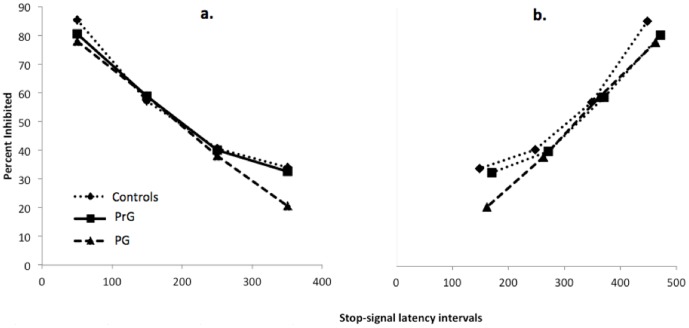
Graphic representation of the proportion of correctly inhibited responses plotted, for each group (controls, PrG, PG), as a function of (**a.**) stop-signal delay (50; 150; 250; 350); (**b.**) the difference between RT for non-stop trials and stop-signal delay. PrG = problem gamblers; PG = pathological gamblers.

Results of the GoStop are presented in Figures 3 and 4. Probability of inhibition was lower for pathological gamblers than control subjects or problem gamblers, but only when the stop-signal delay was long (see Figure 3). A repeated measures ANOVA was performed, with group as a between-subjects factor; stop-signal delay as a within subjects factor; and the proportion of correctly inhibited responses as the dependent measure. This analysis revealed an effect of latency interval, *F*(3, 97) = 239.48, *p*<.001, η^2^ = .71, which is consistent with the race model, and a trend group× latency interval interaction, *F*(6, 94) = 1.84, *p* = .092, η^2^ = .04. To explore the interaction, we conducted pairwise group comparisons for each latency interval conditions. These analyses revealed that PrG (*M* = 36.02; *SD* = 18.79) and normal controls (*M* = 34.04; *SD* = 19.89) performed better than PG (*M* = 23.62; *SD* = 15.84) for SSD = 350 ms, *t*(64) = 2.86, *p*<.01; *t*(62) = 2.19, *p*<.05, respectively. There were no differences for the shorter SSDs.

In addition, we observed no group difference on proportion of novel stimuli no-respond (PG: *M* = 98.67; *SD* = 3.41; PrG: *M* = 97.76; *SD* = 3.25; controls: *M* = 98.57; *SD* = 2.49, *F*(1, 99) = 0.31, ns). We also computed inhibition function plots [for a complete description of this method, see 37] in order to control for the possibility that between-groups differences on stop-signal trials might be due to abnormal reaction time (RT) of PG during the task. Inhibition functions plot the probability of responding [or in our case, probability of inhibiting, which is 1-p(respond)] as a function of stop-signal delay, and have been used frequently to compare inhibitory control in different groups, tasks or conditions [37]. However, group differences in probability of stopping could be caused by differences in reaction times to the target stimuli. We can account for that by plotting probability of stopping as a function of mean RT – SSD. If group differences in probabilities of stopping are indeed due to differences in mean RT, inhibition functions should become aligned. Thus, to explore the inhibition deficit in our study, we plotted for each group the proportion of correctly inhibited responses for (a) stop-signal delays (50; 150; 250; 350); (b) the difference between mean of RT for non-stop trials and stop-signal latency intervals. Figure 4 shows that the misalignment of inhibition function is not modified when RT for non-stop trials was taken into account. This result suggests that between-group differences on the proportion of correctly inhibited responses is related to weak motor response inhibition, and not differences in mean RT. Recent work also suggests that a distinction can be made between proactive inhibition when people anticipate a stop signal to occur, and a fast reactive inhibition process when a signal is presented [39]–[41]. The p(respond) data suggest an impairment in reactive inhibition. A closer inspection of the reaction time data suggest that the subjects in all groups were slower to respond in the second block of the experiment (Controls: *M* = 516.85, *SD* = 133.74; PrG: *M* = 540.73, *SD* = 149.78; PG: *M* = 522.07, *SD* = 91.50); compared with the first block of the experiment (Controls: *M* = 481.00, *SD* = 119.39; PrG: *M* = 502.26, *SD* = 124.02; PG: *M* = 504.18, *SD* = 92.41; block effect: *F*(1,99) = 36.039, *p*<.0001; group effect: *F*(2,98) = 0.33, *p* = .72; group× block interaction: *F*(2,98) = 1.45, *p* = .24). Response slowing could be a marker of proactive inhibition, but see also [41]. Therefore, this finding suggests that there is no impairment in proactive inhibition in pathological gamblers. However, the design was not optimized to study proactive inhibition so future research is required to examine this aspect of behavioral control.

## Discussion

The aim of the present study was to investigate the relationship between the severity of gambling dependence and two facets of impulsivity: *impulsive action* and *impulsive choice*. Impulsive action was assessed using a variant of the stop-signal task; impulsive choice was estimated with the delay-discounting task. In comparison with non-gamblers, non-treatment seeking excessive gamblers were impaired on both *impulsive action* and *impulsive choice*, which is in line with a number of studies that reported impaired pre-potent inhibition performance [7], [15]–[18] and higher rate of discounting [19], [26]–[28], [42] in pathological gamblers (PG). Compared with non-gamblers, problem gamblers (PrG) performed worse in the *impulsive choice* task but not in the *impulsive action* task. Finally, when we compared PG with PrG directly, we found that they performed similarly in the impulsive choice task. By contrast, on the impulsive action task, PG were less efficient than PrG. Specifically, only PG showed impaired performance in the most demanding condition of the stop-signal task (i.e, the 350 ms SSD condition), that is to say, when the stop signal is presented close to the moment of response execution, see race model by Logan and Cowan [35]. When the stop signal delay was short and the response was not prepared yet, PG and their controls performed similarly. Thus, it is especially under conditions in which a response is close to execution and a fast inhibition process is required, that stopping seems to fail in pathological gamblers.

Importantly, all these effects remained after controlling for the influence of a number of possible confounds (anxiety, depression, ADHD and IQ level). Furthermore, our excessive gamblers groups (problematic and pathological) are similar with respect to the duration of gambling experience, which suggests that higher *impulsive action* is a risk factor to become a pathological gambler. In other terms, impulsive action has an impact on the severity of abnormal gambling. By contrast, *impulsive choice* seems to act as general risk factor for abnormal gambling, regardless of the level of severity.

Results regarding the impact of impulsive action on the severity of abnormal gambling are consistent with those of previous studies. For instance, Odlaug and colleagues [18] recently found that PG exhibited significant deficiencies in motor impulse control in the stop-signal task compared with no-risk and at-risk gamblers. Results showing that impulsive choice did not discriminate PrG from PG are consistent with MacKillop and colleagues [28] who highlighted higher rate of discounting in PG and PrG as compared with recreational gamblers. Nevertheless, present results on impulsive choice are also in apparent contradiction with findings from two studies [26], [27] that have highlighted a relation between problem gambling severity and delay discounting. Reasons of the discrepancy between these findings and present results could be due to differences in sample characteristics. Indeed, participants selected for Stea [27] study were mostly recreational or problem (rather than pathological) gamblers. Participants recruited from Alessi and Petry [26] were pathological gamblers enrolled in gambling treatment centers, which contrasts greatly with our sample, that is, gamblers not seeking any treatment. Moreover, there is evidence that impulsive choice, as measured by the DDT, is not static but changes following abstinence from addictive behavior [43].

Collectively, the present results showed that pathological gambling is associated with *impulsive action* and *impulsive choice* whereas problem gambling is only associated with *impulsive choice*. In other terms, these findings suggest that gamblers may manifest impulsivity in fairly specific ways according to their degree of gambling dependence severity. Results of our study are in line with a research from Diergaarde and colleagues [25] in which *impulsive action* and *impulsive choice* predict vulnerability to distinct stages of nicotine seeking behavior in rats. Indeed, impulsive action was primarily associated with higher level of nicotine self-administration whereas impulsive choice was related to an inability to inhibit nicotine seeking during abstinence together with an enhanced sensitivity to nicotine-associated cues. Based on this research, we suggested that problem gambling would be related to an enhanced reactivity towards gambling cues (*impulsive choice*) whereas pathological gambling would be characterized by both enhanced gambling cues reactivity (*impulsive choice*) and inability to disengage from gambling behavior (*impulsive action*). In sum, the present results further support the role of impaired *impulsive action* in severe problem gambling behavior. The main implication of these findings is that intervention strategies aimed at reducing gambling should increase gambler's capacity to disengage from gambling cues or behavior.

A limitation of this study is that we did not recruit gamblers enrolled in a gambling-related treatment program. The present findings are also limited to casino gamblers. Therefore, our conclusions may not apply to pathological gamblers involved in a therapeutic treatment and might not generalize to gamblers with other forms of gambling practices. Finally, we also didn't recruit non-problem gamblers as a control for gambling interest and habit. Hence, “problem gambling” could not be isolated per se. Thus, it is certainly important to extend this research to a larger sample of gamblers, which has both extreme ends of the spectrum of gambling dependence well represented.

Several strengths of the current study are also of note. This research is the first that simultaneously examined the influence of gambling dependence severity on both impulsive action and impulsive choice. Second, the present study employed a three-group design, with a comparison of two groups of gamblers that differ according to the range of problems with gambling. This allowed us to suggest that gamblers may manifest impulsivity in fairly specific ways according to their degree of gambling dependence severity. Third, this study explores impulsivity more accurately by utilizing a task-based approach rather than self-reported measures. Fourth, we recruited control participants with very similar demographic features to gamblers, and the two gamblers groups were also very similar.

To conclude, this study highlighted that *impulsive action* could make the transition between regulated to compulsive gambling faster whereas *impulsive choice* could act as general factor of more elevated risk of excessive gambling. This is important because one could expect that clinical interventions that aim to improve impulse control, and especially impulsive action, might also help to reduce susceptibility to gambling dependence and continued gambling use, or, alternatively lead to successful gambling cessation.
